# Extensive Spinal Disease

**Published:** 1828-05

**Authors:** 


### EXTENSIVE SPINAL DISEASE.

A YOUNG man, twenty-six years of age, born of phthisical parents, and who lost two sisters by pulmonary disease, took to the occupation of chimney sweeper at the age of twelve, and led a drunken and irregular life, sometimes sleeping on wet straw, in barns and out-houses, summer and winter. After a night spent in this way, he experienced some stiffness in the lower extremities, accompanied by weakness. These spread to the arms; and, in the course of a year, he became affected with vertigo in the head, and tremor of the limbs. These incapacitated him for his occupation. One day he fell some yards down a chimney, and cut his head against the iron scraper; but the wound healed without much difficulty. Nevertheless, the trembling and weakness of the limbs increased very much after this accident, and he was obliged to seek refuge in an hospital. When received, he presented the following symptoms: Tremor, and even convulsions (on making any exertions), in the upper extremities; inability to stand or walk without stumbling; heat and sensibility of the surface natural; no pain in any part of the spinal column; constant sense of vertigo; no headache; chest well formed; respiration easy and deep, with the power of lying in any position; all the secretions and excretions natural; appetite good; intellectual faculties weak.

The disease was considered to be effusion into the vertebral canal, with some affection also of the head. A seton was made in the nucha, and various remedies were prescribed, which we shall not here enumerate. Moxas were also applied to the spine. A long and pretty severe course of tartar emetic, however, nearly removed the tremors and vertigo. The patient had got to the quantity of twenty-four grains of tartrite of antimony in the twenty-four hours. But the patient did not gain strength, and he left the hospital uncured.

In July 1826, he had another fall down a chimney; bruised his chest, and spat blood, with cough, pain in the thorax, and other symptoms which led the medical attendants to suspect serious mischief. A violent fever supervened, and again he entered the hospital. On examination, no disease of the lungs could be ascertained, but the heart was found pulsating in the right side of the chest. The expectoration indicated approaching phthisis, and was mixed with blood. After various vacillations, and a variety of remedies directed towards symptoms as they arose, anasarca came on, and the miserable patient sank exhausted on the 16th November, 1826.

*Dissection.*—There were several vesicles filled with fluid between the dura mater and pia mater: the latter membrane was every where thickened and opake; substance of the anterior lobes of the brain remarkably dense; six ounces of clear water in the ventricles; no disease of cerebellum. The spinal marrow was examined with great care. Nothing wrong appeared in its coverings. When these were slit open in front, the spinal marrow bulged out in five different places, giving the appearance of five salient points composed of whiter substance than the rest of the chord. On examining the two superior projections, the structure of the chord was found disorganised, and changed into a substance resembling thick pus. Beneath the third prominence there was found imbedded in the spinal chord a solid body, of a kidney shape, and extremely vascular. A similar substance was discovered in the fourth projection. Between these salient parts, the spinal marrow presented a natural appearance. The lungs were disorganised, partly tuberculous, partly suppurated. The heart and pericardium were unaltered.—*Medico-Chirur. Review.*

---

